# Primary headaches in patients with generalized anxiety disorder

**DOI:** 10.1007/s10194-010-0290-4

**Published:** 2011-02-05

**Authors:** Juliane P. P. Mercante, Mario F. P. Peres, Márcio A. Bernik

**Affiliations:** 1 Anxiety Disorders Program, Institute of Psychiatry, HC-FMUSP, Sao Pãulo, Brazil; 2 Hospital Israelita Albert Einstein, Institute of Teaching and Research, São Paulo, Brazil; 3 Unifesp, EPM, São Paulo, Brazil; 4 Rua Maestro Cardim, 887, Paraíso, São Paulo, SP CEP 01323-001 Brazil

**Keywords:** Migraine, Anxiety disorders, Generalized anxiety disorder, Comorbidity

## Abstract

Although anxiety disorders and headaches are comorbid conditions, there have been no studies evaluating the prevalence of primary headaches in patients with generalized anxiety disorder (GAD). The aim of this study was to analyze the lifetime prevalence of primary headaches in individuals with and without GAD. A total of 60 individuals were evaluated: 30 GAD patients and 30 controls without mental disorders. Psychiatric assessments and primary headache diagnoses were made using structured interviews. Among the GAD patients, the most common diagnosis was migraine, which was significantly more prevalent among the GAD patients than among the controls, as were episodic migraine, chronic daily headache and aura. Tension-type headache was equally common in both groups. Primary headaches in general were significantly more common and more severe in GAD patients than in controls. In anxiety disorder patients, particularly those with GAD, accurate diagnosis of primary headache can improve patient management and clinical outcomes.

## Introduction

Migraine is a common and debilitating chronic neurological illness [1], the lifetime prevalence rates of which are 12–22% in women and 4–10% in men [2]. The prevalence of chronic migraine in the general population ranges from 2 to 3% [3–5]. At tertiary-care headache centers, up to 60% of all consultations are for migraine [6]. In the management of patients with chronic migraine, comorbidity is an important issue. A common comorbidity is anxiety, which is found in up to 75% of cases [7].

The Epidemiologic Catchment Area Study conducted by the United States National Institute of Mental Health from 1980 to 1984, involved 18,500 adults in 5 communities. That study, which provided the most consistent epidemiologic data currently available, demonstrated that anxiety disorders were the most prevalent mental disorders in the general population. Subsequently, the National Comorbidity Survey, conducted in 1990, showed that the lifetime prevalence of anxiety disorder was 24% [8]. In clinical settings, the most common anxiety disorder is generalized anxiety disorder (GAD). In the general population, the lifetime prevalence of GAD is 3.1%, whereas the 12-month prevalence is 5.1% [8].

A high level of disability, a low level of life satisfaction and an impaired quality of life have often been associated with GAD [9], which is also the anxiety disorder most often associated with migraine [10, 11].

To date, there have been no studies using standardized, validated methods of assessing psychiatric diagnosis and psychiatric symptoms in order to evaluate the prevalence of primary headaches in patients with GAD.

Merikangas et al. [11] found GAD incidence to be five times higher in migraineurs (9.8%) than in nonmigraineurs (2.0%). Similar results were obtained for social phobia, which was diagnosed in 6.6% of the migraineurs and 2.0% of the nonmigraineurs. Breslau et al. [12] also found GAD prevalence to be higher among migraine patients than among controls. Anxiety disorders, including phobia, panic disorder, GAD and obsessive–compulsive disorder, were found to be strongly associated with migraine. Both studies [11, 12], however, had methodological limitations; headache diagnosis was not confirmed by a headache specialist, and most of the mental health clinical data was filled out by patients.

In the present study, we analyzed the prevalence of primary headaches in GAD patients.

## Methods

Patients with GAD were recruited from among those voluntarily enrolled in the Anxiety Disorders Program of the Institute of Psychiatry, operating out of the University of Sao Paulo School of Medicine Hospital das Clínicas, where the study was performed. Thirty patients diagnosed with GAD, according to the Diagnostic and Statistical Manual of Mental Disorders, Fourth Edition (DSM-IV), were included in the study; 7 (23.3%) were males, and 23 (76.6%) were females. The mean age of the GAD patients was 42.6 ± 12.2 years (range 19–64 years).

Thirty-two control subjects without mental disorders, participants of an earlier study [13], were submitted to a clinical interview with the author, and 30 were included in the study; 14 (46.6%) were males, and 16 (53.3%) were females. The mean age of the controls was 39.5 ± 11.2 years (range 23–70 years).

All patients were carefully interviewed for psychiatric assessment, using a standardized, validated psychiatric diagnostic method, the Structured Clinical Interview for DSM-IV Axis I Disorders, Patient Edition (SCID-1/P) [14], which was applied by a psychologist with extensive training in clinical psychiatry. Primary headaches were diagnosed according to the criteria established in the Second Edition of International Classification of Headache Disorders (ICHD-II), using a structured interview [15].

All of the patients underwent a clinical interview in order to collect data related to demographics, anthropometrics, headache features, analgesic consumption and psychiatric comorbidities, as well as medical and social consequences. Patient demographic data included gender, age, education level, marital status and employment status. The anthropometric data collected were weight, height and body mass index. In terms of headache-related variables, the lifetime prevalence of primary headaches was evaluated, as was the intensity, duration, frequency and age of onset of headache symptoms. In addition, use of analgesics and consumption of caffeine were quantified. Symptoms were evaluated using the following instruments: the Hamilton Anxiety Rating Scale [16]; the Hamilton Depression Rating Scale [17]; the Fatigue Severity Scale [18]; the Chalder Fatigue Scale [19]; the Visual Analog Scale for Fatigue [20]; and the Epworth Sleepiness Scale [21]. Headache intensity was scored on a scale from 0 to 10. Medical and social consequences, such as headache disability, use of health services and quality of life, were analyzed using the Migraine Disability Assessment questionnaire [22] and the Medical Outcome Study 36-Item Short-Form Health Survey (SF-36) [23].

During the standardized SCID interview, questions regarding the onset of GAD symptoms and the onset of headache symptoms were posed by a single interviewer. The responses to the latter set of questions were confirmed by a neurologist. Only responses in which patients and researchers both presented a significant degree of confidence were included in the analysis. All clinical measures were rated by clinicians and not patients.

Patients presenting medical conditions, mental disorders or situations that might place the patient at significant risk, interfere with results or substantially affect patient participation were excluded.

The study protocol complied with Good Clinical Practice principles and the Helsinki Declaration, and the study design was approved by the local research ethics committees of the institutions involved. All patients gave written informed consent.

The SAS statistical package, version 8.2 (SAS Institute, Cary, NC, USA), and the Minitab statistical package, version 14 (Minitab Inc., State College, PA, USA), were used for statistical analysis. The initial descriptive analysis of variables included frequency, mean and standard deviation. Quantitative data are expressed as mean ± standard deviation. Proportions between two independent groups were compared using the χ^2^ test or Fisher’s exact test when appropriate. The Mann–Whitney nonparametric test was used to compare variables between two independent groups. Linear correlation analysis between two quantitative variables was applied using Pearson’s correlation coefficient (*r*). Coefficients ranged from −1 to 1 and were rated low (for an *r* < 0.4), moderate (for an *r* of 0.4–0.7) or high (for an *r* > 0.7). All significance probabilities (*P* values) shown were two-tailed, and values of *P* < 0.05 were considered statistically significant. Gender was not included in the statistical model, since, given the sample size, we opted for a more conservative strategy involving nonparametric analyses.

## Results

Demographic data for GAD patients and controls are presented in Table 1. No significant differences were found between the two groups in terms of gender, age, level of education, marital status or employment status.

**Table 1:** Demographic characteristics of the individuals studied

Characteristic	GAD (*n* = 30)	Controls (*n* = 30)	*P*
Gender			NS*
Male, *n* (%)	7 (23.3)	14 (46.6)	
Female, *n* (%)	23 (76.6)	16 (53.3)	
Age (years), mean ± SD (range)	42.6 ± 12.2 (19–64)	39.5 ± 11.2 (23–70)	NS**
Education			NS*
0–8 years, *n* (%)	10 (33.3)	7 (23.3)	
9–11 years, *n* (%)	9 (30.0)	6 (20.0)	
≥12 years, *n* (%)	11 (36.7)	17 (56.7)	
Employed, *n* (%)	27 (90)	26 (86.7)	NS*
Marital status			NS*
Married, *n* (%)	14 (46.7)	19 (63.3)	
Unmarried, *n* (%)	16 (53.3)	11 (36.7)	
Divorced, *n* (%)	3 (10)	3 (10)	
Separated, *n* (%)	0 (0.0)	1 (3.3)	
Widowed, *n* (%)	2 (6.7)	0 (0.0)	
Single, *n* (%)	11 (36.7)	7 (23.3)	

*GAD* generalized anxiety disorder (patients)

* χ^2^ test

** Mann–Whitney test

As can be seen in Table 2, there were more women than men in the GAD group. Although this difference was not significant, it might have skewed the results. However, within the GAD group, there were no gender-related differences in terms of the frequency of primary headaches, headache or tension-type headache (TTH).

**Table 2:** Prevalence of primary headache, migraine and tension-type headache in generalized anxiety disorder patients, by gender

Gender	No primary headache	Primary headache	Total	*P*
Male, *n* (%)	1 (14)	6 (86)	7	1.00*
Female, *n* (%)	3 (13)	20 (87)	23	
Total	4	26		

	No migraine	Migraine	Total	
Male, *n* (%)	3 (43)	4 (57)	7	0.858*
Female, *n* (%)	7 (30)	16 (70)	23	
Total	10	20		

	No TTH	TTH	Total	*P*
Male, *n* (%)	5 (71)	2 (29)	7	0.866*
Female, *n* (%)	19 (83)	4 (17)	23	
Total	24	6		

*TTH* Tension-type headache

* Mann–Whitney test

Anthropometric data for GAD patients and controls are presented in Table 3. The two groups presented no significant differences in terms of weight, height or body mass index.

**Table 3:** Anthropometric data

Characteristic	GAD (*n* = 30)	Controls (*n* = 30)	*P*
Weight (kg), mean ± SD	67.28 ± 14.58	71.57 ± 15.21	NS*
Height (m), mean ± SD	1.62 ± 0.09	1.70 ± 0.12	NS*
Body mass index (kg/m^2^), mean ± SD	25.8 ± 5.7	24.4 ± 3.2	NS*
Body mass index ≥25, *n* (%)	14 (46.7)	14 (46.7)	NS**

*GAD* generalized anxiety disorder (patients)

* Mann–Whitney test

** χ^2^ test

Table 4 shows that the lifetime prevalence of primary headaches was higher among GAD patients than among controls (86.7 vs. 46.7%; *P* = 0.001; OR = 7.43; 95% CI = 2.08–26.55). The frequency of migraine was also higher among GAD patients than among controls (66.7 vs. 13.3%; *P* < 0.001; OR = 13.00; 95% CI = 3.55–47.6), episodic migraine (43.3 vs. 10%; *P* = 0.004; OR = 6.88; 95% CI = 1.71–27.75), chronic daily headache (20 vs. 0%; *P* = 0.024) and aura (26.6 vs. 3.3%; *P* = 0.026; OR = 10.55; 95% CI = 1.23–90.67). The prevalence of TTH was comparable between the GAD group and the control group (20 vs. 33.3%; *P* = 0.243).

**Table 4:** Lifetime prevalence of primary headache

Headache type/subtype	GAD, *n* (%)	Controls, *n* (%)	*P*	OR	95% CI
Primary headache	26 (86.7)	14 (46.7)	0.001*	7.43	2.08–26.55
Migraine	20 (66.7)	4 (13.3)	<0.001*	13.00	3.55–47.6
Episodic migraine	13 (43.3)	3 (10.0)	0.004*	6.88	1.71–27.75
Episodic migraine without aura	8 (26.7)	2 (6.6)	0.038*	5.09	0.98–26.43
Episodic migraine with aura	5 (16.6)	1 (3.3)	NS**	5.8	0.63–53.01
Chronic migraine	5 (16.6)	0 (0.0)	0.052**	13.16	0.69–249.19
Chronic migraine without aura	2 (6.6)	0 (0.0)	NS**	5.35	0.25–116.32
Chronic migraine with aura	3 (10)	0 (0.0)	NS**	7.76	0.38–157.15
Probable migraine without aura	2 (6.6)	1 (3.3)	NS**	2.07	0.18–24.15
Chronic daily headache	6 (20.0)	0 (0.0)	0.024**	16.18	0.87–301.64
TTH	6 (20.0)	10 (33.3)	NS*	0.5	0.15–1.62
Infrequent episodic TTH	2 (6.6)	7 (23.3)	NS**	0.23	0.04–1.24
Frequent episodic TTH	3 (10.0)	3 (10.0)	NS*	1	0.19–5.4
Chronic TTH	1 (3.3)	0 (0.0)	NS**	3.1	0.12–79.23
Aura	8 (26.6)	1 (3.3)	0.026***	1.055	1.23–90.67
Primary headache with aura	8 (30.8)	1 (7.1)	0.124***	578	0.64–52.03

Mann–Whitney test

*GAD* Generalized anxiety disorder (patients), *TTH* tension-type headache

* χ^2^ test

** Fisher’s exact test

Primary headache, migraine, episodic migraine and aura exhibited stronger associations with the GAD group than with the control group.

Headache characteristics are presented in Table 5. The frequency of headaches was significantly higher in GAD patients than in controls (9.3 ± 9.5 vs. 2.0 ± 2.0 days per month; *P* = 0.001), as was headache duration (20.9 ± 22.3 vs. 4.5 ± 7.6 h; *P* = 0.004) and headache intensity (5.6 ± 2.2 vs. 3.9 ± 2.4; *P* = 0.049). For these variables, there were no differences between the GAD and control groups in terms of the proportions of men and women.

**Table 5:** Headache characteristics

Headache characteristic	GAD (*n* = 26)	Controls (*n* = 14)	*P*
Frequency (days per month), mean ± SD (range)	9.3 ± 9.5 (1–30)	2.0 ± 2.0 (0.3–6)	0.001**
Intensity (0–10), mean ± SD (range)	5.6 ± 2.2 (2–10)	3.9 ± 2.4 (2–8)	0.049**
Duration (h), mean ± SD (range)	20.2 ± 22.3 (1–72)	4.5 ± 7.6 (0.5–24)	0.004**

*GAD* generalized anxiety disorder (patients)

** Mann–Whitney test

The GAD patients presented higher Hamilton Anxiety Rating Scale scores than did the controls (22.1 ± 6.1 vs. 2.4 ± 2.4; *P* < 0.001). Approximately 40% of the GAD group patients reported moderate to severe anxiety, and 33.3% reported mild to moderate anxiety, whereas all of the controls reported only mild anxiety (Table 6).

**Table 6:** Symptomatology

Instrument	GAD (*n* = 30)	Controls (*n* = 30)	*P*
HAM-A score, mean ± SD (range)	22.1 ± 6.1 (10–34)	2.4 ± 2.4 (0–8)	<0.001*
HAM-D score, mean ± SD (range)	13.7 ± 6.4 (6–28)	1.5 ± 2.5 (0–11)	<0.001*
ESS score, mean ± SD (range)	7.3 ± 6.7 (0–21)	4.2 ± 3.7 (0–14)	NS*
ESS score ≥ 10,* n* (%)	11 (36.7)	2 (6.7)	0.004**
ESS score < 10,* n* (%)	19 (63.3)	28 (93.3)	
FSS score, mean ± SD (range)	47.6 ± 14.9 (9–56)	13.6 ± 14.9 (9–35)	<0.001*
FSS score > 27, n (%)	19 (63.3)	1 (3.3)	0.001**
FSS score < 27, n (%)	11 (36.7)	29 (96.7)	
CFS score, mean ± SD (range)	22.9 ± 7.2 (12–34)	3.5 ± 4.5 (1–19)	<0.001*
VAS-F score, mean ± SD (range)	22.1 ± 31.6 (0–100)	15.0 ± 31.6 (0–70)	<0.001*

*GAD* generalized anxiety disorder (patients), *HAM-A* Hamilton anxiety scale (0–56 points), *HAM-D* Hamilton depression scale (0–50 points), *ESS* Epworth sleepiness scale (0–24 points), *FSS* fatigue severity scale (9–63 points), *CFS* Chalder fatigue scale (0–11 points), *VAS-F* visual analog scale for fatigue (0–100 points)

* Mann–Whitney test

** χ^2^ test

The GAD group patients presented higher Hamilton Depression Rating Scale scores than did the controls (13.7 ± 6.4 vs. 1.5 ± 2.5; *P* < 0.001). Among the GAD patients, mild depressive symptoms were reported by 60%, moderate depressive symptoms by 16.7% and severe depressive symptoms by 10%, whereas 90% of the controls reported no depressive symptoms whatsoever (Table 6).

As can be seen in Table 6, the GAD patients scored higher than did the controls on all three fatigue scales: the Fatigue Severity Scale (47.6 ± 14.9 vs. 13.6 ± 14.9; *P* < 0.001); the Chalder Fatigue Scale (22.9 ± 7.2 vs. 3.5 ± 4.5; *P* < 0.001); and the Visual Analog Scale for Fatigue (22.1 ± 31.6 vs. 15.0 ± 31.6; *P* < 0.001). Fatigue was diagnosed as significantly incapacitating (Fatigue Severity Scale score >27) in 63.3% of the GAD group patients, compared with 3.3% of the controls (*P* = 0.001; OR = 3.45). Table 6 also shows that there was no difference between GAD patients and controls in terms of the mean score on the Epworth Sleepiness Scale (7.3 ± 6.7 vs. 4.2 ± 3.7; *P* = 0.118). Excessive daytime sleepiness (Epworth Sleepiness Scale score ≥10) was diagnosed in 36.7% of the GAD group patients, compared with only 6.7% of controls (*P* = 0.004; OR = 2.13).

Table 7 shows that the Migraine Disability Assessment score was significantly higher for GAD patients with primary headaches than for controls with primary headaches (29.1 ± 68.4 vs. 2.1 ± 0.2; *P* = 0.019), and that GAD patients more often made use of health care services for medical consultations, emergencies or examinations (12.5 ± 8.1 vs. 2.4 ± 2.6 visits/year; *P* < 0.001).

**Table 7:** Medical and social consequences of headache

Variable	GAD	Controls	*P*
MIDAS score, mean ± SD (range)	29.1 ± 68.4 (0–260)	2.1 ± 0.2 (0–3)	0.019**
Use of healthcare services, mean ± SD (range)	12.5 ± 8.1 (0–33)	2.4 ± 2.6 (0–9)	<0.001**
SF-36 functional capacity domain, mean ± SD (total *n*)	65.3 ± 25.1 (*n* = 29)	95.8 ± 5.6 (*n* = 30)	<0.001**
SF-36 physical function domain, mean ± SD (total *n*)	49.1 ± 41.4 (*n* = 29)	97.5 ± 7.6 (*n* = 30)	<0.001**
SF-36 bodily pain domain, mean ± SD (total *n*)	64.1 ± 20.7 (*n* = 29)	88.8 ± 18.8 (*n* = 30)	<0.001**
SF-36 general health domain, mean ± SD (total *n*)	72.1 ± 21.2 (*n* = 29)	92.4 ± 10.6 (*n* = 30)	<0.001**
SF-36 vitality domain, mean ± SD (total *n*)	43.4 ± 21.7 (*n* = 29)	82.0 ± 13.6 (*n* = 30)	<0.001**
SF-36 social function domain, mean ± SD (total *n*)	56.0 ± 31.1 (*n* = 29)	96.7 ± 7.9 (*n* = 30)	<0.001**
SF-36 emotional function domain, mean ± SD (total *n*)	44.8 ± 44.8 (*n* = 29)	97.8 ± 12.2 (*n* = 30)	<0.001**
SF-36 mental health domain, mean ± SD (total *n*)	42.2 ± 22.5 (*n* = 29)	85.2 ± 14.1 (*n* = 30)	<0.001**

*GAD* generalized anxiety disorder (patients), *MIDAS* migraine disability assessment, *SF-36* 36-item short-form health survey

* Mann–Whitney test

The GAD patients scored lower in all eight domains of the SF-36 (i.e., had a poorer quality of life) than did the controls (Table 7).

Consumption of analgesics was higher in the GAD patients than in the controls (2.3 ± 3.3 vs. 0.5 ± 1.2 tablets per week; *P* = 0.031). In terms of the excessive use of analgesics (for more than 15 days), there was no difference between GAD patients and controls (16.7 vs. 3.3%; *P* = 0.645).

The GAD group presented lower caffeine consumption than did the control group (244.14 ± 340.68 vs. 380.74 ± 442.59 mg/day; *P* = 0.042). Excessive consumption of caffeine (>200 mg/day) was more common in the control group than in the GAD group (40 vs. 66.67%; *P* = 0.05).

Of the GAD patients evaluated, 96.6% were diagnosed with at least one psychiatric comorbidity: principally another anxiety disorder (83.33%) or depression (43.33%).

In relation to migraine characteristics, migraine duration presented a positive correlation with the Hamilton Anxiety Rating Scale score and a negative correlation with the score in the vitality domain of the SF-36 (the longer the migraine lasted, the more anxiety symptoms the individual presented and the poorer the quality of life was in terms of vitality). In addition, migraine frequency presented a positive correlation with more frequent use of health care services.

In relation to the progression of GAD, the average age at onset of episodic migraine was higher in the GAD group than in the control group (30.7 ± 14.8 vs. 13 ± 6.8 years; *P* = 0.035). Considering only GAD patients diagnosed with primary headache, the onset of GAD preceded that of episodic migraine in 60% (20.1 ± 13.7 vs. 31.5 ± 14.4 years). The average age at onset of mood disorder was comparable to that of episodic migraine (31.5 ± 14.4 vs. 32.3 ± 13.3; *P* = NS). Of the sample as a whole, 43.3% presented a triple diagnosis: GAD, primary headaches (principally migraine) and depression.

Patients were treated mainly with SSRIs (escitalopram, citalopram), SNRIs (venlafaxine, duloxetine) combined with neuromodulators (topiramate 25–150 mg, divalproate 500–2,000 mg). Patients with sleep problems were administered melatonin 3–12 mg 30 min before bedtime. Tryciclics antidepressants when tolerated were also used. Data about psychopharmachological therapies was included; however, since it was not the main aspect of the research, we could not assess follow-up results and proper clinical endpoints.

## Discussion

There is a lack of data in the literature regarding the prevalence of primary headaches in GAD patients. However, many studies have shown that migraine patients are at increased risk for affective and anxiety disorders [10, 11, 24, 25].

The primary finding of the present study was that the GAD patients differed from the controls in terms of the frequency of migraine headaches but not in terms of the frequency of TTH. This finding is in agreement with the results of previous studies [10, 11] of affective disorders, in which it has been suggested that there is a distinct and bidirectional relationship between affective disorders and migraine, a relationship that does not exist (or is at best unclear) between affective disorders and TTH. The comorbidity between affective disorders and GAD is generally high, and it therefore would not be surprising to find a distinct relationship between GAD and migraine. This might indicate that there is some basic biological disturbance underpinning the interrelationships among depression, anxiety and migraine.

Given the fact that the depression scores were quite high among the GAD patients evaluated in the present study, the data obtained could be interpreted to mean that the GAD patients with migraine were indeed those patients with chronic affective disorders. In fact, this seems not to be the case, since the SCID-1/P interview results indicate that 56.67% of the GAD patients did not suffer from depression, and 43.33% presented major depressive episodes.

Comorbidity between primary headaches and GAD increases the severity of both conditions. The characteristics of primary headache episodes worsened more often in GAD patients than in controls. The severity of symptoms (anxiety, depression, daytime sleepiness and fatigue), as well as the medical and social consequences (functional incapacity, greater use of health care services and lowered quality of life), were also more exacerbated in the GAD patients than in the controls. Other authors have found high headache frequency to be associated with a history of panic disorder [25, 26]. In another study, depression and anxiety disorders were associated with both migraine and nonmigrainous headache, and this association seems more dependent on headache frequency than on diagnostic category [27]. In a general population study, it was found that 59% had headaches with a frequency of 1–4 attacks per month [28, 29]. A nationwide study in France showed that 56% of individuals experienced headaches lasting from 2 to 12 h [29], and that 58–85% had headaches of strong or very strong intensity [29]. In a study of the prevalence and indirect costs of headache in a Brazilian company, the mean frequency of headache was 4.3 ± 7.0 attacks per month, and the mean headache duration was 12.2 ± 21.4 h [30].

Among our GAD patients, the perception of pain intensity during migraine attacks was similar to that found in the literature using the same instruments [28, 30]. However, in the assessment of an extremely subjective phenomenon, methodological bias cannot be ruled out. This difference may be due only to observed differences in the severity of headaches.

We found that comorbidity with primary headaches had a negative impact on several measures of symptom severity and quality of life in individuals with GAD. Our results indicate severe functional disability and a marked personal burden (anxiety, depressive symptoms, fatigue and excessive daytime sleepiness), as well as medical and social consequences (functional incapacity, greater use of healthcare services and lowered quality of life), in patients with primary headache and concomitant GAD.

The consumption of analgesics was greater in the GAD patients with primary headache than in the controls with primary headache. This finding is also in accordance with those of other studies [31]. The ingestion of analgesics can occur prior to the onset of a headache, due to anxiety and excessive worrying, which are characteristic of GAD. Ferrari [32] appraised reasons patients gave for this behavior: 67% reported difficulty in coping with the pain, 62% feared its emergence, and 45% consumed analgesics to reduce anxiety.

Another possibility is that of higher caffeine consumption rebound headaches [33] and generalized anxiety in GAD patients. However, caffeine consumption was higher in the controls.

In individuals presenting more symptoms of anxiety, migraines lasted longer, lending credence to the hypotheses that GAD patients create an attention bias for headaches, and this heightened perception can effectively lengthen the duration of the headaches. In addition, individuals experiencing migraines more frequently also make more frequent use of health care services, which is also related to excessive, difficult to control worrying, which is typical of GAD. World Health Organization data suggest that GAD is the most common anxiety disorder in primary care.

Our finding that 96.6% of GAD patients were diagnosed with at least one psychiatric comorbidity—principally another anxiety disorder (83.33%) or depression (43.33%)—is in accordance with the literature, which indicates that untreated GAD leads to long-term complications, such as the development of major depressive disorder [34, 35].

## Disease progression

In terms of age at onset, GAD preceded episodic migraines. The onset of episodic migraine age tended to occur late in the GAD group than in the controls, suggesting that migraines are a long-term consequence of untreated GAD.

Nearly half (43.3%) of our patients presented a triple diagnosis: GAD, primary headaches and depression.

These findings are consistent with the progressive disorder model proposed by Merikangas et al. [11], in which anxiety disorders generally precede migraine, which is then followed by depression. The authors postulated that there is “a syndromic relationship between migraine, anxiety and depression”, a spectrum of symptoms initiating with anxiety (frequently in early infancy), followed by the occurrence of migraines and subsequent depressive episodes in adulthood.

The fact that, in the present study, the onset of episodic migraines tended to occur later in the GAD patients than in the controls might indicate that the headaches experienced by GAD patients were secondary to anxiety.

## Comorbidity

Longitudinal studies have shown a specific bidirectional association between migraine and depression, suggesting a common etiology. There are no definitive data that explain the association between GAD and migraine. Environmental or genetic risk factors might produce a brain state that predisposes to both conditions, i.e., there might be some common biology underlying the two conditions [36]. There appears to be an association between migraine and affective disorders, particularly depression and anxiety. Migraine and other primary headaches heighten the risk of onset/aggravation of GAD, whereas GAD heightens the risk of onset of migraine and other primary headaches. The latter effect seems to be more consistent, since the onset of GAD occurs at an earlier age than does that of episodic migraine.

The apparently distinct and bidirectional relationship that we found between affective disorders and migraine, a relationship that does not exist (or is at best unclear) between affective disorders and TTH, is in line with the results of previous studies analyzing affective disorders. The comorbidity between affective disorders and GAD is generally high, and it therefore would not be surprising to find a distinct relationship between GAD and migraine. This might indicate that there is some basic biological disturbance underpinning the interrelationships among depression, anxiety and migraine.

A recent paper [37] was published looking at 206 consecutive outpatients in an anxiety disorders clinic sample, the prevalence of migraine was 67%. The severity of anxiety disorder symptoms was significantly higher in patients with migraine compared with patients without migraine.

## Limitations

The present study had some methodological limitations.

The small sample size (*n* = 30) and the especially small number of males (*n* = 7) in the GAD sample create a number of problems: lack of statistical power can lead to the false conclusion that differences in some variables do not exist (Type II error), whereas the performance of a number of statistical tests that is greater than the number of subjects in a group (without correction for the number of tests) can lead to chance findings (Type I error). However, in observing GAD group migraine frequency, we found no gender-related differences in terms of diagnoses of primary headache, headache or TTH. The small number of males limits the ability to test for gender-related differences, which might exist for some variables.

Another limitation of the study was the fact that the control group was composed not of ‘normal’ individuals (general population) but of ‘supernormal’ individuals (proven to be free of psychiatric disorders). In addition, the appraiser could not be blinded as to the presence or absence of a psychiatric diagnosis, since symptoms were reported during the neurological interview.

Finally, there was the limitation in relation to causality. Further longitudinal studies could provide more information on the course and progression of GAD.

## Conclusion

Strengths of this manuscript include the reporting of data for a carefully diagnosed sample of patients with GAD and the use of standardized, validated methods of assessing psychiatric diagnosis and psychiatric symptoms.

Our results indicate that the high prevalence of primary headaches in GAD patients has a significant impact on the lives of those patients and on the health care system. Diagnosing primary headache is important in patients with anxiety disorders, particularly those with GAD, since correct assessment can lead to better patient management and more favorable clinical outcomes.
